# Impact of antiretroviral therapy on clinical outcomes in HIV ^+^ kidney transplant recipients: Review of 58 cases

**DOI:** 10.12688/f1000research.10414.1

**Published:** 2016-12-21

**Authors:** Rossana Rosa, Jose F. Suarez, Marco A. Lorio, Michele I. Morris, Lilian M. Abbo, Jacques Simkins, Giselle Guerra, David Roth, Warren L. Kupin, Adela Mattiazzi, Gaetano Ciancio, Linda J. Chen, George W. Burke, Jose M. Figueiro, Phillip Ruiz, Jose F. Camargo

**Affiliations:** 1 Department of Medicine, Jackson Memorial Hospital, Miami, USA; 2 UnityPoint Health, Des Moines, USA; 3 Department of Medicine, Division of Infectious Diseases, University of Miami Miller School of Medicine, Miami, USA; 4 Department of Medicine, Division of Nephrology, University of Miami Miller School of Medicine, Miami, USA; 5 Department of Surgery, University of Miami Miller School of Medicine and Miami Transplant Institute at the Jackson Memorial Hospital, Miami, USA

**Keywords:** HIV, kidney transplant, protease inhibitor, antiretroviral therapy, infection

## Abstract

*Background:* Antiretroviral therapy (ART) poses challenging drug-drug interactions with immunosuppressant agents in transplant recipients.  We aimed to determine the impact of specific antiretroviral regimens in clinical outcomes of HIV ^+^ kidney transplant recipients. *Methods:* A single-center, retrospective cohort study was conducted at a large academic center. Subjects included 58 HIV ^-^ to HIV ^+^ adult, first-time kidney transplant patients. The main intervention was ART regimen used after transplantation.  The main outcomes assessed at one- and three-years were: patient survival, death-censored graft survival, and biopsy-proven acute rejection; we also assessed serious infections within the first six months post-transplant. *Results:* Patient and graft survival at three years were both 90% for the entire cohort. Patients receiving protease inhibitor (PI)-containing regimens had lower patient survival at one and three years than patients receiving PI-sparing regimens: 85% vs. 100% ( *p*=0.06) and 82% vs. 100% ( *p*=0.03), respectively. Patients who received PI-containing regimens had twelve times higher odds of death at 3 years compared to patients who were not exposed to PIs (odds ratio, 12.05; 95% confidence interval, 1.31-1602; *p*=0.02).  Three-year death-censored graft survival was lower in patients receiving PI vs. patients on PI-sparing regimens (82 vs 100%, *p*=0.03). Patients receiving integrase strand transfer inhibitors-containing regimens had higher 3-year graft survival. There were no differences in the incidence of acute rejection by ART regimen. Individuals receiving PIs had a higher incidence of serious infections compared to those on PI-sparing regimens (39 vs. 8%, *p*=0.01). *Conclusions:* PI-containing ART regimens are associated with adverse outcomes in HIV ^+^ kidney transplant recipients.

## Introduction

More than 500 kidney transplants in human immunodeficiency virus–infected (HIV ^+^) recipients have been performed in the United States with acceptable outcomes ^1– 5^. HIV infection is associated with a two- to three-fold increase in the risk of rejection ^3^. Reduced exposure to immunosuppressive agents is considered the main mechanism for increased predisposition to rejection ^3, 6, 7^.

Drug-drug interactions between antiretroviral therapy (ART) and calcineurin inhibitors (CNI), such as tacrolimus, pose a significant clinical challenge. Protease inhibitors (PI) and cobicistat increase the levels of CNI, whereas nonnucleoside reverse transcriptase inhibitors (NNRTI) reduce the levels of these agents. In contrast to PI and NNRTI, integrase strand transfer inhibitors (INSTI), which are not a substrate of CYP450, have become the preferred antiretroviral in many centers to overcome the problematic pharmacokinetic interactions ^6– 9^.

Although tenofovir disoproxil fumarate (TDF) has a good safety profile and is recommended as a first-line agent ^10^, it can cause renal tubular dysfunction in HIV ^+^ individuals ^11^ and tenofovir-related nephrotoxicity is always a concern in kidney transplant recipients.

Data on the impact of specific ART regimens on the clinical outcomes of HIV ^+^ kidney transplant recipients is scarce. In the present study, we compared post-transplant outcomes by ART regimens in a group of 58 HIV ^+^ kidney recipients transplanted at our institution over a 9-year period.

## Methods

### Study subjects

A single-center, retrospective cohort study of 58 consecutive HIV ^-^ to HIV ^+^ adult, first-time kidney transplants performed in the Miami Transplant Institute affiliated to Jackson Memorial Hospital, a 1,550-bed academic medical center, between October 2006 and October 2015. All HIV ^+^ recipients had an undetectable viral load, and all but one (a kidney-liver recipient) had a CD4 count > 200 cells/mm ^3^ at the time of the transplant. The study was approved by the University of Miami institutional review board (#20150614). Written consent was waived by the institutional review board due to the retrospective observational nature of the study.

### Immunosuppression protocol

Immunosuppression and antimicrobial prophylaxis protocols at our center have been previously described ^4, 5^.

### Clinical outcomes

The one- and three-year outcomes assessed were: patient survival, death-censored graft survival, and biopsy-proven acute rejection; we also assessed serious infections within the first six months post-transplant, defined as infections requiring admission to the intensive care unit during initial transplant hospitalization or re-admission to the hospital after discharge ^4^.

### Statistics

The Fisher exact test and Wilcoxon Mann–Whitney U test were used where appropriate. Univariate analyses were performed using logistic regression with penalized likelihood estimation. Multivariable models were not pursued due to small number of events. Log-rank test was used to assess differences in time-to-event. Statistical analyses were performed using SAS University Edition (SAS Institute Inc., Cary, NC, USA).

## Results

### Patient characteristics

A total of 58 HIV ^+^ adult kidney allograft recipients were studied ( Table 1). In total, 51 subjects had at least one HIV viral load during the first year post-transplant, and except for six patients who had transient “blips” in viremia (median peak viremia, 130 copies/mL [IQR, 114–193]), all the patients had sustained ART-induced HIV viral load suppression (<50 copies/mL) post-transplant.

**Table 1.** Baseline characteristics of study participants ^*^.

Variable	All patients n=58 (%)	Protease inhibitor		*P*-value
		PI-sparing regimen n=25 (%)	PI-containing regimen n=33 (%)	
***Demographics***				
Age, median (IQR)	48 (43-54)	49 (43-55)	47 (42-49)	0.13
Age, older than 40	45 (78)	20 (80)	25 (76)	0.70
Male gender	38 (66)	15 (60)	23 (70)	0.44
African-American	43 (74)	20 (80)	23 (70)	0.37
***HIV infection***				
Pre-transplant CD4 count <350 cells/mm ^3^	16 (28)	7 (28)	9 (27)	0.95
Pre-transplant CD4 count, cells/mm ^3^, median (IQR)	504 (351-666)	441 (362-648)	579 (346-666)	0.54
Pre-transplant CD4/CD8 ratio, median (IQR)	0.7 (0.6-1)	0.7 (0.6-0.8)	0.7 (0.5-1.1)	0.67
Time from HIV diagnosis, years, median (IQR)	10 (5-16)	12 (7-17)	10 (5-15)	0.24
***Comorbidities***				
Hepatitis C	7 (12)	1 (4)	6 (18)	0.13
Diabetes mellitus	11 (19)	8 (33)	3 (9)	0.04
Hypertension	38 (66)	19 (58)	19 (76)	0.14
HIVAN	41 (72)	17 (71)	24 (73)	0.87
Overweight (BMI >25)	28 (48)	13 (52)	15 (45)	0.79
***Immunosuppression ^†^***				
Prednisone	52 (90)	24 (96)	28 (85)	0.22
IVIG	5 (9)	0	5 (15)	0.06
Rituximab	4 (7)	1 (4)	3 (9)	0.63
Tacrolimus	57 (98)	25 (100)	32 (97)	>0.99
MMF	57 (98)	25 (100)	32 (97)	>0.99
Sirolimus	3 (5)	0	3 (9)	0.25
Cyclosporine	2 (3)	2 (8)	0	0.18
***Kidney allograft***				
Post-transplant follow-up, years, median (IQR)	1.8 (0.9-4.2)	1.7 (1-4.8)	1.9 (0.9-3.5)	0.40
Transplant year 2006–2010	34 (59)	16 (64)	18 (55)	0.59
Living donor	14 (24)	6 (24)	8 (24)	0.98
Donor age, median (IQR)	39 (28-47)	45 (29-47)	37 (21-47)	0.42
Delayed graft function	6 (11)	1 (4)	5 (15)	0.38
Cold ischemia time, >36h ^Ϯ^	9 (19)	3 (13)	6 (24)	0.47
HLA-ABDR mismatches, >5 ^Ϯ^	21 (39)	8 (38)	13 (39)	>0.99
ABC-PRA, <5%	48 (92)	20 (91)	28 (93)	>0.99
DR-PRA, <5%	49 (92)	19 (86)	30 (97)	0.30
CMV viremia ^Ϯ^, >500 copies/mL	2 (4)	0	2 (6)	0.51
BK viremia ^˄^, >10,000 copies/mL	5 (10)	3 (14)	2 (7)	0.65

BMI, body mass index; CMV, cytomegalovirus; HIV, human immunodeficiency virus; HIVAN, HIV-associated nephropathy; IQR, interquartile range; IVIG, Intravenous immunoglobulin; MMF, mycophenolate mofetil; PRA, panel reactive antibody. PI, protease inhibitor. ^*^Data presented as absolute number (percentage), unless specified otherwise. The *p*-value corresponds to comparison of PI-containing and PI-sparing groups by using the Fisher exact test. Wilcoxon Mann–Whitney test was used for variables presented as median and IQR. ^†^All of the patients received anti–thymocyte globulin, basiliximab and methylprednisolone for induction. ^Ϯ^Cold ischemia time and HLA-mismatch data available for 47 and 54 patients, respectively. ^Ϯ^During first year post-transplant.

### Antiretroviral therapy

There were no ART restrictions in transplant eligibility for HIV ^+^ candidates during the study period. The three most common regimens post-transplant were nucleoside reverse transcriptase inhibitors (NRTI) plus PI, NRTI plus INSTI, and NRTI plus NNRTI ( Table 2).

**Table 2.** Distribution of ART regimens among 58 HIV ^+^ kidney transplant recipients.

ART regimen	Pre-transplant	Post-transplant ^†^	Post-transplant (12 months)
	n (%)	n (%) ^*^	n (%) ^º^
***Single drug class combination***			
NRTI	1 (2)	1 (2)	1 (2)
***Two drug class combination***			
NRTI + PI	30 (52)	23 (40)	11 (27)
NRTI + INSTI	2 (3)	12 (21)	8 (20)
NRTI + NNRTI	15 (26)	9 (16)	7 (17)
PI + INSTI	4 (7)	2 (3)	2 (5)
NNRTI + INSTI	1 (2)	1 (2)	0
NNRTI + PI	0	0	1 (2)
***Three drug class combination***			
NRTI + PI + INSTI	2 (3)	6 (10)	4 (10)
NRTI + PI + NNRTI	2 (3)	1 (2)	0
NRTI + INSTI + NNRTI	1 (2)	2 (3)	4 (10)
NNRTI + INSTI + PI	0	1 (2)	2 (5)
***Four drug combination***			
NRTI + INSTI + NNRTI + PI	0	0	1 (2)

ART, antiretroviral therapy; INSTI, integrase strand transfer inhibitors; NRTI, nucleoside reverse transcriptase inhibitors; NNRTI, nonnucleoside reverse transcriptase inhibitors; PI, protease inhibitors. ^†^Refers to the ART regimen the patient was discharged home with after the initial transplant hospitalization. ^º^Data only available for 41 patients (due to death, loss of follow up, or insufficient documentation in medical record). ^*^Individual percentage values are rounded and might not total 100%.

**Figure 1.**
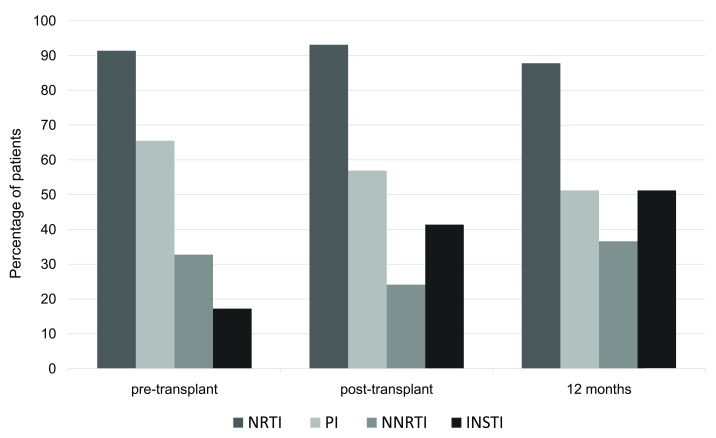
Frequency of HIV ^+^ kidney transplant recipients receiving a given ART class in the pre-transplant (n=58), post-transplant (at time of discharge; n=58) and at 12 months post-transplant follow-up (n=41). There was a significant increase in the proportion of patients receiving INSTI-containing regimens at time of discharge and 12 months post-transplant. ART, antiretroviral therapy; INSTI, integrase strand transfer inhibitors; NRTI, nucleoside reverse transcriptase inhibitors; NNRTI, nonnucleoside reverse transcriptase inhibitors; PI, protease inhibitors.

A total of 30 (52%) patients underwent ART modifications after transplant; 22 (38%) of them prior to discharge, and an additional 8 (14%) during the first year post-transplant. Adjustments in ART were primarily done to avoid drug-drug interactions or added nephrotoxicity. There was a significant increase in the proportion of patients receiving INSTI at time of discharge and at 12 months post-transplant compared to pre-transplant period: 41% ( *p*<0.01) and 51% ( *p*<0.0005) vs. 17%, respectively ( Table 2 and Figure 1).

### Transplant outcomes by ART regimen

**Figure 2.**
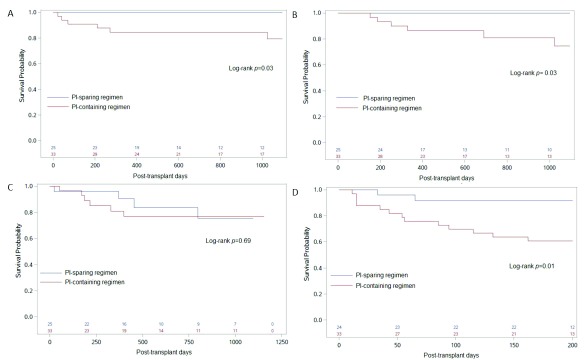
Transplant outcomes in HIV ^+^ kidney transplant recipients by administration of protease inhibitor (PI) at time of discharge. Kaplan–Meier curves show the ( **A**) 3-year patient survival, ( **B**) 3-year graft survival, ( **C**) 3-year rejection-free survival, and ( **D**) 200-day infection-free survival in PI-sparing (blue) and PI-containing (red) groups. The number of patients in each group is shown in the bottom of each panel.

The patient and graft survival at three years were both 90% for the entire cohort. Transplant outcomes varied by ART regimen at the time of discharge after the initial transplant hospitalization. Patients receiving PI-containing regimens had lower patient survival at one and three years than patients receiving PI-sparing regimens: 85% vs. 100% ( *p*=0.06) and 82% vs. 100% ( *p*=0.03), respectively ( Table 3 and Figure 2). Patients who received PI-containing regimens had twelve times higher odds of death at three years compared to patients who were not exposed to PIs (odds ratio [OR] 12.05; 95% confidence interval [CI] 1.31-1602; *p*=0.02). Hepatitis C and delayed graft function also increased the odds of death, but this finding did not reach statistical significance ( Table 4). Three-year death-censored graft survival was lower in patients receiving PI vs. patients on PI-sparing regimens (82 vs 100%, *p*=0.03; Table 3 and Figure 2). On the contrary, patients receiving INSTI-containing regimens had higher three-year graft survival rates (100 vs. 82%, *p*=0.04; Table 3).

**Table 3.** One and three-year transplant outcomes by ART regimen ^°^.

	N (%)	Patient survival				Death-censored graft survival ^†^				Biopsy-proven acute rejection				Severe infection ^†^	
		1y	*p*	3y	*p*	1y	*p*	3y	*p*	1y	*p*	3y	*p*	6m	*p*
Overall	58	91.4		89.7		93.1		89.7		13.8		17.2		26.3	
TDF
Yes	19	89.5		89.5		94.7		94.7		15.8		10.5		31.6	
No *	35	94.3	0.6	91.4	>0.99	91.4	>0.99	85.7	0.4	14.3	>0.99	22.9	0.4	23.5	0.52
NRTI
Yes	54	92.6		90.7		92.6		88.9		14.8		18.52		26.4	
No	4	75	0.3	75	0.36	100	>0.99	100	>0.99	0	>0.99	0	>0.99	25	
NNRTI
Yes	14	100		100		92.9		92.9		7.14		21.4		7.14	
No	44	88.6	0.32	86.4	0.32	93.2	>0.99	88.6	>0.99	15.9	0.66	15.9	0.69	32.6	0.08
PI
Yes	33	84.5		81.8		87.9		81.8		21.2		18.2		39.4	
No	25	100	0.06	100	**0.03**	100	0.12	100	**0.03**	4	0.12	16	>0.99	8.33	**0.01**
INSTI
Yes	24	95.8		95.8		100		100		8.33		8.33		21.7	
No	34	88.2	0.39	85.3	0.38	88.2	0.13	82.4	**0.04**	17.7	0.45	23.5	0.17	29.4	0.5
Two drug regimens ˆ
NRTI + INSTI	12	100		100		100		100		8.3		8.3		18.1	
NRTI + NNRTI	9	100		100		100		100		0		11.1		0	
NRTI + PI	23	82.6	0.16	78.3	0.1	86.9	0.41	78.3	0.1	21.7	0.36	21.7	0.65	39.1	0.07
															
NRTI + other *	21	100		100		100		100		4.8		9.5		10	
NRTI + PI	23	82.6	0.11	78.3	**0.05**	86.9	0.23	78.3	**0.05**	21.7	0.19	21.7	0.42	39.1	**0.04**

ART, antiretroviral therapy; INSTI, integrase strand transfer inhibitors; NRTI, nucleoside reverse transcriptase inhibitors; NNRTI, non-nucleoside reverse transcriptase inhibitors; PI, protease inhibitors; TDF, tenofovir disoproxil fumarate. ^**º**^Refers to the ART regimen the patient was discharged home with after the initial transplant hospitalization. *P* values correspond to Fisher 's exact test. Numbers in bold represent statistical significance. ^**†**^As defined previously ^4^. See main text for details. ˆRegimens listed here were three most common ART regimens post-transplant in this cohort. *Includes NRTI + INSTI and NRTI + NNRTI.

**Table 4.** Variables associated with three-year mortality.

Variable ^Ϯ^	Alive at 3-years n=52 (%)	Death at 3-years n=6 (%)	Odds ratio (95% CI)	*P*-value ^*^
Protease inhibitor use	27 (51.9)	6 (100)	12.1 (1.31-1602)	0.02
HCV co-infection	5 (9.62)	2 (33.3)	4.80 (0.70-28.3)	0.10
Tacrolimus levels at 4 weeks, median (IQR)	6 (4.1-8.5)	8.7 (5.9-11.9)	1.06 (0.91-1.20)	0.38
Recipient age, years, median (IQR)	48 (42-54)	48 (46-49)	1 (0.92-1.10)	0.91
Baseline CD4 <350 cells/mm ^3^	15 (28.9)	1 (16.67)	0.66 (0.06-3.70)	0.66
Delayed graft function	4 (7.84)	2 (33.3)	5.86 (0.84-36.70)	0.07
Type 2 diabetes	11 (21.6)	6 (100)	0.27 (0.002-2.60)	0.31
Donor age, years, median (IQR)	40 (29-47)	24 (18-48)	0.95 (0.88-1.02)	0.16
Living donor	14 (26.9)	0	0.20 (0.002-1.92)	0.19
Time from HIV diagnosis, years, median (IQR)	10 (5-16)	12 (5-13)	0.98 (0.86-1.09)	0.72
Morbid obesity	9 (17.3)	0	0.35 (0.003-3.45)	0.43

^Ϯ^Data presented as absolute number (percentage), unless specified otherwise. * *P*-value calculated using logistic regression with penalized likelihood estimation (null hypothesis of beta=0). HIV, human immunodeficiency virus; IQR, interquartile range.

**Figure 3.**
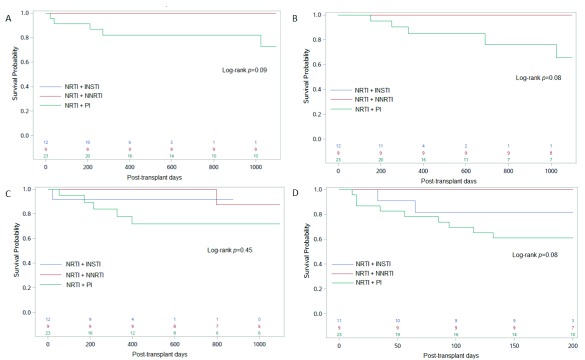
Transplant outcomes in HIV ^+^ kidney transplant recipients by ART regimen at time of discharge. Kaplan–Meier curves show the ( **A**) 3-year patient survival, ( **B**) 3-year graft survival, ( **C**) 3-year rejection-free survival, and ( **D**) 200-day infection-free survival in NRTI + INSTI (blue), NRTI + NNRTI (red) and NRTI + PI (green) groups. Number of patients in each group is shown in the bottom of each panel. ART, antiretroviral therapy; INSTI, integrase strand transfer inhibitors; NRTI, nucleoside reverse transcriptase inhibitors; NNRTI, nonnucleoside reverse transcriptase inhibitors; PI, protease inhibitors.

We next assessed transplant outcomes in patients receiving NRTI “backbone” combined with either NNRTI, PI or INSTI as a second drug class. Compared to a group of patients receiving NRTI plus INSTI or NRTI plus NNRTI, the 3-year patient and graft survival were lower in patients receiving NRTI plus PI (78 vs. 100%; *p*=0.05, Table 3 and Figure 3).

Causes of graft loss among patients on PI-containing regimens were acute rejection in two (33%), thrombosis/hemorrhagic complications in two (33%), CNI toxicity in one (17%), and unidentified in another patient. The cumulative incidence of biopsy-proven acute rejection was 14 and 17% at one and three years post-transplant, respectively. There were no significant differences in rejection rates by ART ( Figure 2 and Figure 3; Table 3).

### Incidence of serious infections by ART

Serious non-opportunistic infections within six months post-transplant occurred in 15 (26%) patients. The etiology of such infections, mainly bacterial and fungal in nature, has been reported previously ^4^. In total, 13 (87%) of these patients were on PI-containing regimens. Individuals receiving PI had a higher incidence of serious infections compared to those on PI-sparing regimens (39 vs. 8%, *p*=0.01; Figure 2). This association remained significant in analyses restricted to patients on NRTI “backbone”: 39 vs. 10% for patients receiving NRTI + PI compared to those receiving NRTI + INSTI or NNRTI, respectively ( *p*=0.04; Table 3 and Figure 3).

### ART and tacrolimus levels

Tacrolimus levels at 4, 12, 26 and 52 weeks post-transplant were within therapeutic range for most patient groups ( Table 5). Although we did not observe differences in tacrolimus levels by ART at these specific time points, out of 11 patients with tacrolimus levels available at the time of infection, six (54%) had supra-therapeutic levels (median, IQR: 9.2, 5.5-10.1).

**Table 5.** Plasma tacrolimus levels by ART regimen.

	*n*	Tacrolimus levels								
		4 weeks	*p*	12 weeks	*p*	26 weeks	*p*	52 weeks	*p*
**Overall**	58	6.1 (4.1-8.5)	-	6.3 (5.3-7.9)	-	6.2 (4.8-8.3)	-	6.2 (5.1-7.8)	-
**TDF**									
**Yes**	19	6.3 (5-8.5)	0.88	6.7 (5.9-7.8)	0.25	6.1 (4.5-8.1)	0.74	6.1 (5.8-7.8)	0.44
No ^*^	35	6.1 (4-9)		6.2 (4.8-7.9)		6.2 (5.1-8.3)		6.2 (4.4-7.6)	
**NRTI**									
**Yes**	54	6.3 (4.4-8.5)	0.23	6.3 (5.3-7.8)	0.51	6.1 (4.7-7.6)	0.03	6.2 (5.2-7.7)	0.82
**No**	4	2.2 (0-17.2)		8 (2.1-15)		9.3 (8.3-19.1)		10.9 (3.9-17.9)	
**NNRTI**									
**Yes**	14	5.9 (4.5-8.3)	0.81	5.8 (4.8-7.1)	0.08	6.2 (5.5-6.5)	0.88	6.3 (5-7.6)	0.99
**No**	44	6.2 (4.1-8.7)		6.4 (5.6-8.2)		6.4 (4.7-8.7)		6.1 (5.3-7.8)	
**PI**									
**Yes**	33	6.6 (4.4-9)	0.41	6.1 (5.3-8.4)	0.42	6.2 (4-9.3)	0.81	6.4 (4.8-8.3)	0.64
**No**	25	5.6 (4-8.4)		6.3 (5-7.4)		6.2 (5.1-7.5)		6.1 (5.6-7.5)	
**INSTI**									
**Yes**	24	4.6 (3.6-7.2)	0.01	7.8 (6.8-9.2)	0.62	7.5 (5.4-9.3)	0.07	6.2 (5.8-7.6)	0.73
**No**	34	5.7 (4.1-7.1)		5.9 (5.1-7.9)		6 (4.4-7.2)		6.1 (4.7-8.3)	
**NRTI + INSTI**	12	4.4 (3.5-7.5)	0.08	6.8 (5.3-7.8)	0.41	6.7 (5.5-7.6)	0.53	6.1 (5.9-7)	0.84
**NRTI + NNRTI**	9	7.7 (5.6-9.7)		5.9 (4.8-7.1)		6.1 (5-6.4)		5.8 (5-7.6)	
**NRTI + PI**	23	7.6 (5.7-10.9)		6.1 (5.7-8.4)		5.9 (3.5-7.5)		6.6 (5.3-8.3)	
**NRTI + other**	21	5.6 (4-8.7)	0.17	6.5 (5-8.1)	0.35	6.3 (5.5-7.5)	0.49	6 (5.6-7.6)	0.60
**NRTI + PI**	23	7.6 (5.7-10.9)		6.1 (5.7-8.4)		5.9 (3.5-7.5)		6.6 (5.3-8.3)	

ART, antiretroviral therapy; INSTI, integrase strand transfer inhibitors; NRTI, nucleoside reverse transcriptase inhibitors; NNRTI, non-nucleoside reverse transcriptase inhibitors; PI, protease inhibitors. TDF, tenofovir disoproxil fumarate. *Only includes patients on NRTI other than TDF. The *p*-value corresponds to comparison of PI-containing and PI-sparing groups by using the Fisher exact test. Tacrolimus target levels at our center are 6–8 ng/mL during the first three months and 5–7 ng/mL after three months post-transplant. Higher levels are targeted for highly sensitized patients.

Rosa et al. Impact of ART in KT outcomes in HIV recipients: Raw data Click here for additional data file. Copyright: © 2016 Rosa R et al. 2016 Data associated with the article are available under the terms of the Creative Commons Zero "No rights reserved" data waiver (CC0 1.0 Public domain dedication).

## Discussion

Consistent with previous studies of kidney transplantation in HIV ^1– 5^, we observed excellent transplant outcomes without evidence of HIV disease progression. The most important finding of the present study is the association between PI use and adverse outcomes, namely reduced three-year patient and graft survival, and increased risk of serious non-opportunistic infections. These observations remained true in analyses restricted to patients receiving NRTI “backbone”; thus, even after excluding the potential influence of other agents included in the ART regimen, PI continued to be associated with poor outcomes. The immunosuppression protocol at our institution remained constant during the study period, and the proportion of patients transplanted in the 2006–2010 (and consequently the 2011–2015) eras was similar between PI and non-PI groups, suggesting that this observation was also independent of variation in transplant practices over time that might have impacted outcomes.

Biopsy-proven acute rejection and CNI toxicity accounted for half of the cases of graft loss in patients taking PI in the present study. Increased risk of allograft rejection in HIV ^+^ individuals has been largely attributed to reduced exposure to immunosuppressive agents, due to drug-drug interactions with ART ^3, 6, 7^. However, in this small cohort, we did not observe an association between ART regimens and the incidence of rejection. CNI levels at 4, 12, 26 and 52 weeks were comparable across ART groups. Other factors, such as infection of the allograft, previous alloimmunization and immune activation, might also play a role in predisposition to rejection ^3, 5, 6^.

Non-opportunistic infections within six months post-transplant are common in HIV ^+^ kidney recipients ^3^, especially those with marginal pre-transplant CD4 counts ^4^. Notably, the occurrence of serious infections in this cohort was almost five-fold higher in patients receiving PI.

This might be due to the effects of PI on tacrolimus levels, considering that the overwhelming majority of these patients were on PI-containing regimen and more-than-half had tacrolimus levels above target at the time of infection. PI could also influence the net state of immunosuppression by increasing the level or effect of other immunosuppressants, such as prednisone and mycophenolate.

Contrary to our expectations, the use of NNRTI or TDF did not influence kidney allograft survival. Tenofovir alafenamide (TAF) is a new formulation of tenofovir associated with less kidney (and bone) toxicity ^12^. Whether there is added clinical benefit of TAF over TDF in kidney transplant recipients remains to be established.

Consistent with recent reports ^7– 9^, patients receiving INSTI-containing regimens had excellent patient survival (96%) and graft survival (100%) at three years, and the lowest rejection rates in this cohort (8%). Current guidelines recommend the use of NRTI plus INSTI as a first-line therapy for HIV ^10^. INSTI pose no interactions with CNI or mTOR inhibitors. In addition, INSTIs have no interactions with direct-acting antivirals, which is important in the setting of hepatitis C co-infection, as that has been associated with poor outcomes ^2, 3^. Thus, it has become our practice to preemptively switch HIV ^+^ candidates pre-transplant or in the immediate post-transplant period to PI-sparing, preferably INSTI-based, ART regimens.

Although none of the patients studied here was on cobicistat, it is important to highlight that this pharmacokinetic enhancer, contained in several combination pills, can increase the levels of CNI ^13^. HIV ^+^ recipients and their community HIV providers should be educated about what ART medications to avoid, and when not possible, how to adjust CNI doses and monitor levels accordingly.

Our study is limited by the small number of patients and retrospective design; serum levels for other immunosuppressants, such as mycophenolate were not available. The association found in the present study between PI-containing ART regimens and adverse outcomes needs to be confirmed in larger studies. Until more data becomes available, the use of PI-sparing regimens in HIV ^+ ^kidney recipients seems to be the most prudent approach.

## Data availability

The data referenced by this article are under copyright with the following copyright statement: Copyright: © 2016 Rosa R et al.

Data associated with the article are available under the terms of the Creative Commons Zero "No rights reserved" data waiver (CC0 1.0 Public domain dedication). 

**Dataset 1: Rosa *et al.* Impact of ART in KT outcomes in HIV recipients: Raw data. doi,** 10.5256/f1000research.10414.d146717 ^14^.
