# Supplementary material for: Impact of antiretroviral therapy on clinical outcomes in HIV + kidney transplant recipients: Review of 58 cases
Source: F1000Res. 2016 Dec 21;5:2893. [Version 1] doi: 10.12688/f1000research.10414.1 (PMC5310378; doi:10.12688/f1000research.10414.1)
Supplement: Peer review history

# Peer review: Referee response for version 1

**DOI:** 10.5256/f1000research.11220.r20186

**Published:** 2017-02-14

**Authors:** Kalathil K Sureshkumar

**Affiliations:** 1 Division of Nephrology and Hypertension, Department of Medicine, Allegheny General Hospital, 320 East North Avenue, Pittsburgh, PA, 15212, USA

This manuscript is a retrospective single- canter analysis of the outcomes of kidney transplantation in HIV infected recipients stratified by anti-retroviral therapy (ART) used.

 The authors found inferior 3 year graft and patient survivals in patients on protease inhibitor (PI) containing regimens. Six month opportunistic infections were also high in patients on PI-containing regimens. ART with integrase inhibitors was associated with better outcomes. Integrase inhibitors such as raltegravir has minimal drug-drug interactions and are being increasingly used in ART regimens.

 Overall, the manuscript is written well and conclusions are supported by the study findings.

 I have following comments:

1. Was there any difference in outcomes based on induction agents used?
2. Any postulations as to why the observed inferior graft and patient outcomes in the PI group other than drug-drug interactions?

I have read this submission. I believe that I have an appropriate level of expertise to confirm that it is of an acceptable scientific standard.

# Peer review: Referee response for version 1

**DOI:** 10.5256/f1000research.11220.r19166

**Published:** 2017-01-26

**Authors:** Merceditas Villanueva

**Affiliations:** 1 Department of Internal Medicine, Section of Infectious Diseases, School of Medicine, Yale University, New Haven, CT, USA

The paper entitled" Impact of antiretroviral therapy on clinical outcomes in HIV+kidney transplant recipients: Review of 58 cases" by Camargo *et. al*. is a well-done study with contributions from a multi-disciplinary group of medical providers (Internal Medicine, Infectious Disease, Nephrology, Surgery). The findings add to a growing experience on the optimal management of HIV+ renal transplants including use of ART. Specifically, it provides statistical analysis demonstrating that PI-containing regimens are significantly associated with worse 3 year survival, 3 year graft function and severe infection rates at 6 months in a relatively large group of patients at a single transplant center.  The results add further weight to our own findings at our center ^1^ about the preferential use of INSTI regimens in this population preferentially used pre-emotively before transplant.

 The study design is well explained and tables and figures are clear.  The conclusions are balanced and justified on the basis of the data.

 My main question is that the outcomes are based on the ART regimen in the immediate post-transplant period (there were 33 patients on PI-containing regimen).  However, the authors state that at 12 months post-transplant, of their available data, only 21 were on PI-containing regimen, due to switches made, in part due to concerns for drug-drug interactions.  So the conclusions regarding 3 year patient and graft survival may not reflect an on-treatment analysis. In other words, at 3 years, how many patients were still actually taking PIs?

 A few other questions to clarify the study would be helpful:

 1) There were 6 deaths at 3 years:  what were the causes of death?  Infections? How many were on PIs at time of death?

 2) What were the specific antiretrovirals used? How was dosing altered post transplant?

 3) Can the authors speculate on what other mechanisms might explain why PI use (aside from effect on CNI or mTOR levels) could affect patient and graft survival?

I have read this submission. I believe that I have an appropriate level of expertise to confirm that it is of an acceptable scientific standard.

## References

[1] AzarMMMalinisMFMossJFormicaRNVillanuevaMS: Integrase strand transferase inhibitors: the preferred antiretroviral regimen in HIV-positive renal transplantation. *Int J STD AIDS*.2016; 10.1177/0956462416651528 10.1177/0956462416651528 27193421
